# Pretreatment of Japanese cedar by ionic liquid solutions in combination with acid and metal ion and its application to high solid loading

**DOI:** 10.1186/s13068-014-0120-z

**Published:** 2014-08-20

**Authors:** Kazuma Ogura, Kazuaki Ninomiya, Kenji Takahashi, Chiaki Ogino, Akihiko Kondo

**Affiliations:** Department of Chemical Science and Engineering, Graduate School of Engineering, Kobe University, 1-1 Rokkodaicho, Nada-ku, Kobe, 657-8501 Japan; Institute of Nature and Environmental Technology, Kanazawa University, Kakuma-machi, Kanazawa, 920-1192 Japan

**Keywords:** Japanese cedar, Pretreatment, Ionic liquid, Cellulose rich material, Lignin, High solid loading

## Abstract

**Background:**

Lignocellulosic biomass from plant biomass, especially softwoods, are well-known to present difficulties during attempts at hydrolysis due to their rigid structure. Pretreatment of lignocellulosic biomass with ionic liquids (ILs) is attractive as this requires to a low input of energy. However, IL pretreatment has the disadvantage of the presence of large amounts of water. Recently, it was reported that a small amount of acid has a positive effect on the degradation of biomass in IL with water. In this study the pretreatment of Japanese cedar, the most abundant softwood in Japan, was investigated using a combination of IL, acid and metal ions.

**Results:**

First, the novel ionic liquid pretreatment was investigated by changing the pretreatment solvent and the anti-solvent. A mixture of IL, acid and ferric oxide (Fe^3+^) ion was most effective for pretreatment, and an acetone-water mixture was also most effective on the precipitation of biomass. These optimized pretreatment combinations attained a higher degree of glucose release from the pretreated biomass. The amount of cellulose was concentrated from to a level of 36 to 84% of the insoluble fraction by the optimized pretreatment. Based on this result, it was assumed that the extraction of the lignin fraction from the biomass into an anti-solvent solution was attained. Finally, this optimized pretreatment was applied to the enzymatic hydrolysis of Japanese cedar at high-solid biomass loading, and 110 g/L of glucose production was attained. In addition, the ethanol fermentation with this hydrolyzed solution by *Saccharomyces cerevisiae* achieved 50 g/L ethanol production, and this yield reached 90% of the theoretical yield.

**Conclusions:**

We developed an effective pretreatment protocol by changing to a pretreatment solvent containing IL, acid, metal ion and anti-solvent. The optimized pretreatment has an effect on softwood and separately retrieved lignin as a by-product. The saccharified solution at high-solid biomass loading was converted to ethanol in a high yield. This proposed methodology would boost the performance of the bioconversion of low-cost materials to other chemicals, and would not be limited to only ethanol but also would include other target chemicals.

## Background

### Utilization of lignocellulosic biomass

Biomass is one of the alternative candidates to replace fossil resources as a source of fuels and valuable chemicals [1–3]. For example bioethanol from starch, so-called first-generation ethanol, already contributes large amounts of liquid fuel in several countries. However, concerns have been widely expressed that the production of fuels from edible biomass directly competes with food production.

Lignocellulosic forms of biomass such as waste wood and agricultural residues have gained attention as renewable and nonedible sources of fermentable sugars for bioconversion into biofuels and biochemical products. Bioethanol from lignocellulosic biomass, so-called second-generation ethanol, shows some advantages compared to first-generation ethanol. Lignocellulosic biomass does not compete directly with crops grown for food, thus the efficiency of land use can be increased. In addition, the huge availability of lignocellulosic biomass makes it suitable for the large-scale manufacture of energy and chemicals. However, this resource has a disadvantage with respect to saccharification. Lignocellulosic biomass is difficult to hydrolyze to a monosaccharide due to its rigid structure. The main components of lignocellulosic biomass are cellulose, hemicellulose and lignin. Cellulose chains are embedded in the core of the lignocellulosic biomass with hydroxyl groups oriented to form strong intra- and intermolecular hydrogen bonds. In addition, lignin is characterized as an amorphous and covalent cross-linked phenylpropanoid polymer. Therefore, these molecular interactions define a complex and rigid structure forming a material that is recalcitrant to hydrolysis [4].

To solve this problem, lignocellulosic biomass must be pretreated prior to the addition of cellulase in order to accomplish the saccharification of cellulose. Therefore, the conversion of lignocellulosic biomass into ethanol and other valuable chemicals generally consists of the following steps: (1) pretreatment to enhance the enzymatic digestibility of lignocellulosic biomass, (2) enzymatic hydrolysis of pretreated biomass to fermentable sugars and (3) assimilation of the sugars to ethanol or other metabolites by microbe fermentation.

Among these steps, the pretreatment of lignocellulosic biomass (first step) is an important unit operation because it greatly affects the efficiency and methodology of the subsequent saccharification process. To overcome the recalcitrance of lignocellulose, various pretreatment methods have been attempted. These pretreatments can be classified into physical [5], physicochemical [6], chemical [7] and biological [8] methods or combinations thereof [9–11]. Among these technologies, steam explosion and dilute sulfuric acid pretreatment are practical and effective methods for disrupting the hydrogen bonds in the crystalline cellulose and the covalent cross-linkages in lignin [12,13]. However, high temperature and high pressure are required for these pretreatments. To reduce total energy consumption, the development of new pretreatment methods is required.

### Ionic liquid and its application to the bioprocess

Ionic liquids (ILs) are salts that are liquid at temperatures of less than 100°C. They have unique beneficial properties, such as negligible vapor pressure, high thermal and chemical stability and the ability to dissolve various polymeric compounds under mild conditions.

In 2002, 1-butyl-3-methylimidazolium chloride ([BMIM] Cl) was first used to dissolve cellulose with no derivatization [14], which suggested new solvent systems for cellulose. Later, some ILs were used to dissolve lignocellulosic biomass at relatively low temperatures, and also were used to regenerate biomass using an anti-solvent [15]. Those applications resulted in significantly accelerated hydrolysis when using cellulases compared with un-pretreated biomass. Among these, 1-ethyl-3-methylimidazolium acetate ([EMIM] OAc) effectively solubilizes biomass, decreases the crystallinity of cellulose and rejects lignin from the recovered polysaccharides [16,17].

However, moisture sensitivity is a major drawback of IL pretreatment [14]. The presence of water in biomass could markedly reduce not only the solubility of cellulose, but also the pretreatment effectiveness. This is a major disadvantage for industrial applications because moisture must be excluded from all the reagents and equipment involved in all operations, and biomass contains significant quantities of water (between 2 and 300%) relative to the oven-dried weight. In addition, ILs are hygroscopic and tend to absorb significant quantities of moisture when exposed to air [18]. These characteristics make ILs difficult to handle and are generally considered to be technical weaknesses that hamper the recyclability of the solvent. To avoid this problem, the drying of biomass and ILs requires heat and a vacuum process for dehydration. Therefore, this process currently is not effective from the standpoint of energy consumption, which confers great importance to the development of moisture-tolerant and highly effective IL pretreatments.

### Acid-catalyzed process in ionic liquid

Recently, it was reported that a small amount of acid has a positive effect on the degradation of biomass in the presence of ILs and significant amounts of water [19–21]. Existing research has dictated that biomass pretreatments required the selective removal of lignin and hemicellulose from cellulose, in other words, ‘biomass fractionation’. However, these results combining IL and acid mainly focused on the dissolution of biomass and cellulose, which is not adequate for pretreatment. In addition, that previous work only focused on the pretreatment of bagasse, which is a herbaceous biomass that has a weak structure compared with the more-reluctant forms of biomass, such as wood. Thus, solvents and anti-solvents offer improvements for wood biomass pretreatment.

Based on a previous report (pretreatment solvent: (IL:hydrochloric acid (HCl):water = 78.8:1.2:20 wt%), anti-solvent: water) [19], we examined ways to improve the pretreatment effectiveness of IL on biomass that has a significant amount of water. For this experiment we used Japanese cedar, which is one of the most abundant softwoods in Japan, and is also one of the most challenging forms of lignocellulosic biomass. First, we constructed a novel IL pretreatment by changing the pretreatment solvent and the anti-solvent. Second, we analyzed the compositional changes through pretreatment by measuring the cellulose and lignin contents in the biomass. Finally, we constructed a bioprocess for bioethanol production with high-solid biomass loading by using biomass with our developed pretreatment in order to test the practical effectiveness.

## Results and Discussion

### Aqueous ionic liquid pretreatment solvent with two catalysts

Acid-catalyzed pretreatment in IL has usually been combined with at least one kind of acid for improvement [19–21]. On the other hand, in the case of diluted acid pretreatment for the biomass in a water system, several studies have shown that inorganic salts also indicate the possibility of improvement for the hydrolysis rate of the hemicellulose and cellulose in biomass [22,23]. In detail, Yan *et al.* investigated the effect of ferric oxide (Fe^3+^) ions on the HCl hydrolysis of peat [23]. Nguyen and Tucker used a dilute acid pretreatment with a mixture containing aqueous solutions of a dilute acid and a metal salt catalyst for hydrolyzing cellulose and hemicellulose in lignocellulosic biomass [22]. This method enabled them to obtain a higher overall fermentable sugar than when using a dilute acid alone. Although these studies have reported the use of two catalysts in a water system [22,23], there has been no report on these applications to pretreatment in IL. Therefore, we investigated the pretreatment effect by using a two-catalyst system (HCl and metal salt) in IL for the pretreatment of biomass. We chose HCl as the acid because a previous study had reported the use of HCl for biomass pretreatment [19]. Furthermore, we tried to use another previously reported catalyst for biomass or lignin deconstruction, and prepared five reagents as a second catalyst: aluminium chloride (AlCl_3_), iron chloride (FeCl_3_), iron sulfate (FeSO_4_), sulfuric acid (H_2_SO_4_) and manganese (II) nitrate (Mn (NO_3_)_2_) [19,24,25], respectively. The pretreatments were conducted in the solvent mixture (20 wt% water, 1.2 wt% HCl, 78.8 wt% [BMIM] Cl and metal salt), and the amount of additional metal salt was adjusted to 7.40 × 10^−2^ mmol in each case (since the weights of each of the additional metal salts were small, the weight of [BMIM] Cl in each combination was almost the same at more than 77.0 wt%).

**Figure 1:**
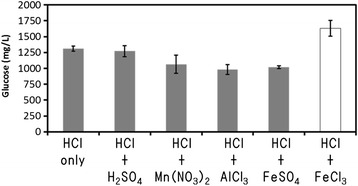
**Effect of the second catalyst in IL on glucose concentration after enzymatic saccharification.** Pretreatment conditions: [BMIM] CI solution containing 20% water and 1.2% HCI, at 103°C for 30 minutes. Each data were averages of three independent experiments ± SD.

The use of FeCl_3_ as the additional metal salt catalyst indicated a higher amount of glucose than the [BMIM] Cl/HCl system (IL:HCl:water = 78.8:1.2:20 wt%; Figure 1). All other metal salt additions had no positive effect on the biomass pretreatment. The reason was that Fe^3+^ enhanced the acid activity. Fe^3+^ seemed to have a strong catalytic effect on the pretreatment process during the acid catalysis with IL. The pretreatment using FeCl_3_ was the most effective compared with the other reagents, so we decided to use FeCl_3_ (7.40 × 10^−2^ mmol = 12 mg) as the additional catalyst in IL.

**Figure 2:**
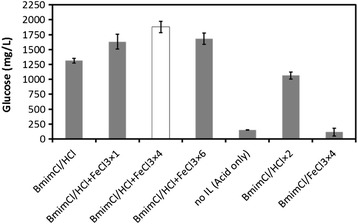
**Effect of FeCI**_**3**_ **amount (12 mg, 48 mg, 72 mg) on Japanese cedar glucose digestibility.** Each data were averages of three independent experiments ± SD.

Encouraged by these results, the amount of FeCl_3_ loading in pretreatment solvent was investigated. By changing the Fe^3+^ loading, the following three pretreatment conditions were compared (Figure 2): [BMIM] Cl/HCl + FeCl_3_ × 1 system: (IL:HCl:FeCl_3_:water = 77.6:1.2:1.2:20 wt%), [BMIM] Cl/HCl + FeCl_3_ × 4 system: (IL:HCl:FeCl_3_:water = 74.0:1.2:4.8:20 wt%), and [BMIM] Cl/HCl + FeCl_3_ × 6 system: (IL:HCl:FeCl_3_:water = 71.6:1.2:7.2:20 wt%). The highest degradation profile was observed in 48 mg of FeCl_3_. In addition, no IL system (H_2_O:HCl:FeCl_3_ = 94:1.2:4.8 wt%) exhibited an effect on biomass deconstruction. This result indicated that acid pretreatment in IL was superior to conventional acid pretreatment in water. According to previous study [21], below the glass transition temperature of lignin (<160°C), a digestible biomass was slightly produced in the absence of IL. Therefore, we believe the absence of an IL caused the low production of glucose. Moreover, the result of a [BMIM] Cl/HCl × 2 system (IL:HCl:water = 77.6:2.4:20) as a control was less than that of a [BMIM] Cl/HCl system. It was assumed that a much higher degree of HCl loading would reduce the sugar yield because the excessive HCl loading would cause the formation of enzyme non-digestive lignin [26].

Based on the above results, we selected the [BMIM] Cl/HCl + FeCl_3_ × 4 system (IL:HCl:FeCl_3_:water = 74.0:1.2:4.8:20 wt%) as the pretreatment solvent for use in further experimentation.

### Regeneration process and the combination of two solvents

The solubilized biomass in ILs can be regenerated by the precipitation from solutions with anti-solvents such as water, acetone and alcohols. In general, the regeneration of biomass for enzymatic hydrolysis via the simple addition of water has been reported. Therefore, the selection of an anti-solvent for IL pretreatment was a critical factor in the fractionation of lignocellulosic biomass [27].

Some researchers have observed that the cellulose content in the precipitate can be enriched when organic solvent mixtures are used instead of pure water in order to keep the lignin apart from the biomass in the solution. For example, an acetone-water mixture (1:1 vol%) was used by Sun *et al*. [16], while Viell and Marquardt used an ethanol-acetone mixture (1:1 vol%) [28]. Wang *et al.* also observed that the addition of dimethyl sulfoxide (DMSO) to water as an anti-solvent was effective in increasing the cellulose content in regenerated biomass [29]. These results showed that the anti-solvent was important for biomass fractionation (lignin separation from biomass). It also affected saccharification, because lignin has a role as the essential glue in a biomass structure and inhibits the biomass degradation activity of cellulose, and closely related non-specific enzymes adsorbed in the saccharification process [30]. Therefore, the selection of suitable anti-solvents in order to enhance the acid-catalyzed pretreatment in ILs was investigated.

**Figure 3:**
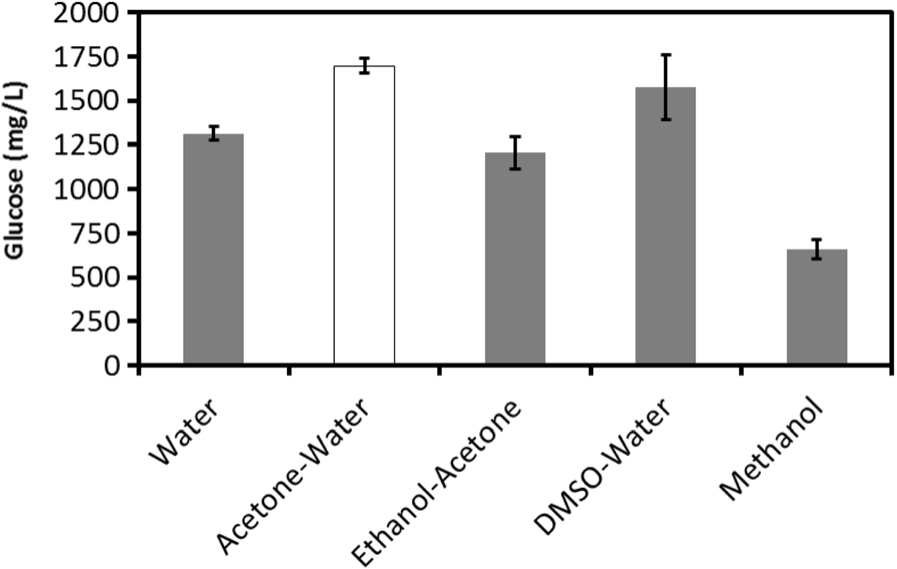
**Effect of the different precipitants from [BMIM] CI/HCI on Japanese cedar glucose digestibility.** Each data were averages of three independent experiments ± SD.

First, the combination of the anti-solvents used in pretreatment [BMIM] Cl/HCl system (IL:HCl:water = 78.8:1.2:20) was evaluated (Figure 3). Several of the anti-solvents mentioned above (acetone-water, ethanol-acetone, DMSO-water and methanol) were prepared, and the selection of an anti-solvent for efficient saccharification was performed (Figure 3). As result, the acetone-water mixture proved to be the most effective for saccharification, while the other two anti-solvents (ethanol-acetone and DMSO-water) indicated negative effects for the saccharification of the pretreated biomass. The differences in the saccharification results may be attributed to differences in the biomass, IL and acid selected. Thus, it was assumed that the selection of the regeneration solvent was a key factor affecting subsequent saccharification.

Second, the combination of the [BMIM] Cl/HCl + FeCl_3_ × 4 system as a pretreatment solvent (IL:HCl:FeCl_3_:water = 74.0:1.2:4.8:20) and acetone-water as an anti-solvent was investigated for the achievement of high glucose production. Four pretreatment combinations were designed based on the above results (Table 1). As a control for comparison, a single pretreatment with [EMIM] OAc was also performed. This IL is well-known as a general solvent for the preparation of biomass, and both lignin and hemicellulose could be partially extracted during pretreatment with [EMIM] OAc [16,17,31].

**Table 1:** **Pretreatment scheme in combination of pretreatment**

**Pretreatment scheme**	**Pretreatment solvent**	**Anti-solvent**
Entry 1 (control)	[BMIM] Cl/HCI	Water
	(IL:HCI:water = 78.8:1.2:20 wt%)	
Entry 2	[BMIM] Cl/HCI + FeCI_3_	Water
	(IL:HCI:FeCI_3_:water = 74.0:1.2:4.8:20 wt%)	
Entry 3	[BMIM] Cl/HCi	Acetone-water
	(IL:HCI:water = 78.8:1.2:20 wt%)	
Entry 4	[BMIM] CI/HCI + FeCI_3_	Acetone-water
	(IL:HCI:FeCI_3_:water = 74.0:1.2:4:20 wt%)	

**Figure 4:**
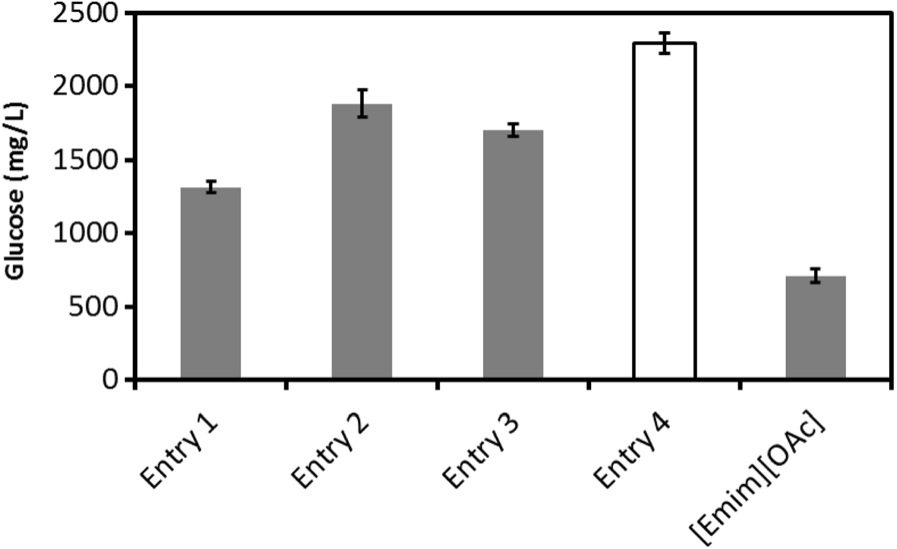
**Effect of anti-solvent (acetone-water) and pretreatment solvent (IL:HCI:FeCI**_**3**_ **:water = 74.0:1.2:4.8:20) and their combination pretreatments on saccharide formation after 24 hours.** Each data were averages of three independent experiments ± SD.

The combination of pretreatment and precipitation was more effective for biomass pretreatment than that of simple pretreatment with [EMIM] OAc and precipitation with water (Figure 4). In comparison with Entry 1, the combination system (Entry 4) resulted in 74% more glucose production. Entry 4 consisted of a pretreatment solvent ([BMIM] Cl/HCl + FeCl_3_ × 4) and an anti-solvent (acetone-water), which resulted in the most effective saccharification to glucose, surpassing that of pretreatment using [EMIM] OAc.

### Influence of temperature on saccharification

**Figure 5:**
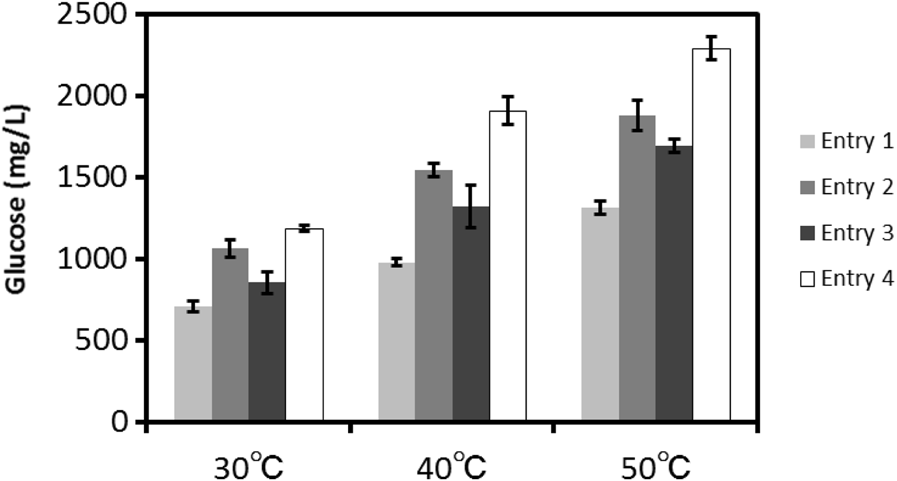
**Saccharification of pretreatment biomass by CTec2 at different temperatures (30°C, 40°C and 50°C).** Each data were averages of three independent experiments ± SD.

Simultaneous saccharification and fermentation (SSF) has proven to be an economically favorable operation compared with separate hydrolysis and fermentation (SHF) in terms of lower energy consumption and maintenance cost, due to the non-inhibition from saccharide. However, the SSF process still reduces the activity of cellulases due to reactions at low temperature. In general, saccharification with cellulolytic enzymes is usually operated at around 50°C. Most fermenting microbes, however, have an optimum temperature for ethanol fermentation of between 30 and 37°C. Therefore, saccharification is usually performed at low temperatures (30 or 37°C) in order to adjust the growth of the microbes. For this purpose, the saccharification of pretreated biomass with an optimized combination of three temperatures at less than 50°C was demonstrated with Cellic CTec2 as a cellulase mixture (Figure 5). As result, the hydrolyzed glucose from pretreated biomass was increased as the reaction temperature was increased from 30 to 50°C in every pretreatment combination. Among them, the optimized combination (Entry 4) was most effective for biomass degradation to glucose in every reaction temperature. In particular, the degradation yield by Entry 4 at 30°C exhibited was almost equal to that of Entry 1 at 50°C, and it was assumed that by using Entry 4 ethanol fermentation by the SSF process from pretreated biomass could be achieved more effectively at 30°C.

### Compositional analysis and material balance of pretreated biomass

Compositional analysis is important for the investigation of pretreatment factors; solid yield (wt%) after pretreatment, composition of treated solids and mass balance were investigated.

**Figure 6:**
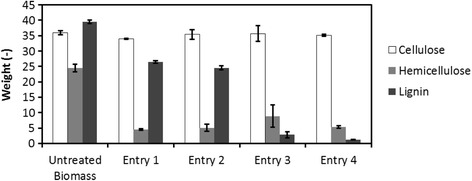
**Compositional change based on 100% untreated biomass weights through each pretreatment.** Each data were averages of two independent experiments.

To investigate the alternations in composition during pretreatment, the following three factors were measured: regenerated biomass, cellulose recovery and lignin recovery (Table 2). Comparisons of the biomass compositions of each pretreatment combination based on 100% untreated biomass weights were also investigated (Figure 6). Cellulose content accounted for 36 wt% of untreated biomass, which agrees with the material information (cellulose fraction weight: 36.72 wt%). Based on the results shown in Table 2 and Figure 6, the combination (Entry 4) could be a novel pretreatment for the following three reasons: (1) the cellulose recovery rate amounted to more than 97% of the untreated biomass; (2) almost all lignin was removed compared with that of the other entries; and (3) the cellulose content of pretreated biomass was increased from 36 to 84%. Based on these results, it was assumed that the Entry 4 pretreatment could convert lignocellulosic biomass to cellulose-rich material. The cellulose content of the pretreated biomass in Entry 4 was similar to that obtained by Kraft pulping (90%), which implied the possibility of a reduction of energy consumption in pulping using IL.

**Table 2:** **Various pretreatment factors about composition**

**Pretreatment scheme**	**Regenerated biomass (%)** ^**1**^	**Cellulose recovery (%)** ^**2**^	**Lignin recovery (%)** ^**3**^	**Cellulose content (%)** ^**4**^
Untreated	100.0	100.0	100.0	36.0
Entry 1	64.9 ± 0.3	94.61 ± 0.4	66 ± 1.0	52.4 ± 1.6
Entry 2	65.1 ± 1.1	98.5 ± 4.5	62.2 ± 1.6	54.4 ± 1.6
Entry 3	47.4 ± 2.9	99.3 ± 7.2	7.2 ± 2.5	75.3 ± 5.5
Entry 4	41.8 ± 1.1	97.8 ± 0.9	3.1 ± 0.2	84.2 ± 0.8

Each data were averages of two independent experiments ± SD.

^1^Regenerated biomass = (mass of dried and regenerated biomass)/(mass of initial dried biomass) × 100 (%).

^2^Cellulose recovery = (mass of cellulose in dried and regenerated biomass)/(mass of initial cellulose) × 100 (%).

^3^Lignin recovery = (mass of lignin in dried and regenerated biomass)/(mass of initial dried lignin) × 100 (%).

^4^Cellulose content = (mass of cellulose in dried and regenerated biomass)/(mass of dried and regenerated biomass) × 100 (%).

Acetone-water as an anti-solvent (in Entry 4) was a good solvent for lignin extraction. Lignin recoveries in Entries 1 and 2 (anti-solvent:water) were more than 60%, but those in Entries 3 and 4 (anti-solvent:acetone-water) were less than 10%. Therefore, the precipitation process by anti-solvent (acetone-water) was assumed to be critical for removal of the lignin from biomass, because the lignin fraction was well-known to be an obstacle to the saccharification of biomass.

**Figure 7:**
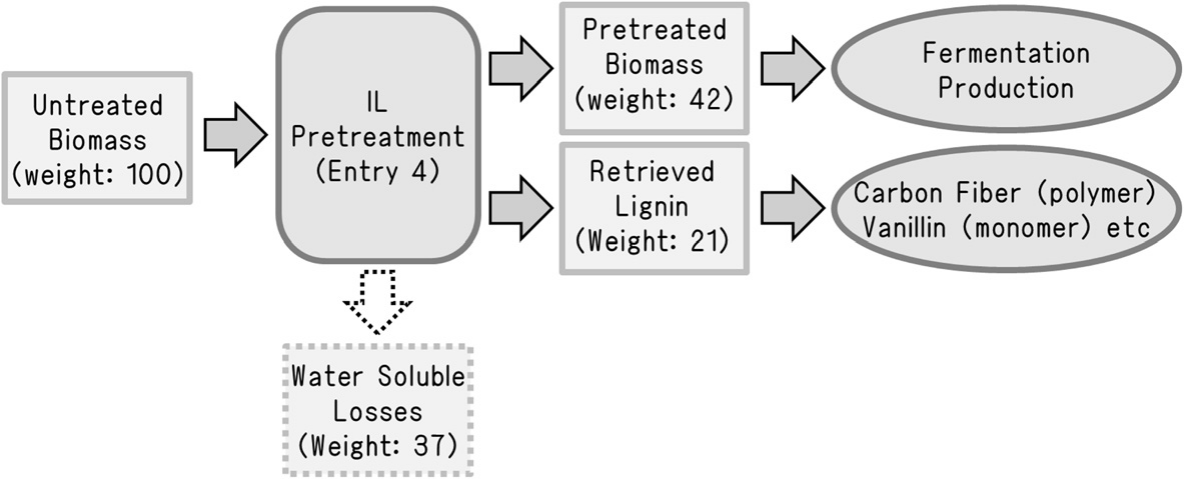
**Separation of the two components of lignocellulose using novel pretreatment protocol and perspective of ligin utilization.**

Lignin can be retrieved from the anti-solvent solution after the evaporation of acetone and water [16,32]. Therefore, the collection of lignin as a by-product of our novel pretreatment process (Entry 4) was performed. Acetone in the anti-solvent solution was evaporated at 60°C. The resultant material balance scheme for the pretreatment process is summarized in Figure 7. The lignin in the acetone-water was retrieved easily after the evaporation of the acetone. The retrieved lignin fraction was expected to be approximately 21 wt% of the original biomass content (or 54 wt% of the original lignin content). Sun *et al.* isolated 10 wt% of lignin from small particles of pine [16]. Cox and Ekerdt recovered 20 wt% of lignin from yellow pine wood chips by using an acidic IL [32]. However, these pretreatments required a high temperature and a long treatment time (150°C and 75 minutes at least). We succeeded in recovering more than 20% of lignin under comparable mild pretreatment conditions (130°C and 30 minutes). This material could be a promising resource for carbon fiber (polymer), vanillin (monomer) and other products [33,34]. The present study was focused on not only the cellulose fractionation, but also the extraction of lignin, and it was also important in terms of effective total utilization of biomass.

**Figure 8:**
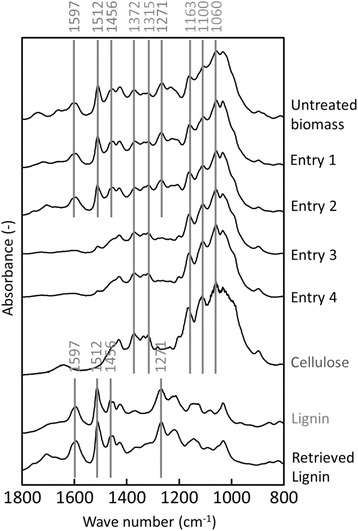
**FTIR spectra of biomass sample.** Control regent (cellulose and lignin) is purchased from Sigma-Aldrich (St. Louis, Missouri, United States). Vertical lines denote the characteristic peaks of cellulose and lignin.

Finally, we conducted Fourier Transform Infrared Spectroscopy (FTIR) analysis of pretreated biomass to confirm the fractionation of biomass. Based on the peaks related to cellulose/hemicellulose and lignin, the composition of biomass could be analyzed [35–37]. Some peaks were responsible for cellulose and hemicellulose: 1372 cm^−1^ (C–H bending vibration), 1315 cm^−1^ (O–H, C–C, C–O), 1163 cm^−1^ (C–O–C asymmetric bridge stretching vibration), 1100 cm^−1^ (O–H association band) and 1060 cm^−1^ (C–O stretching vibration). Other peaks belonged to lignin: 1597 cm^−1^ (aromatic skeletal vibration breathing with C = O stretching), 1512 cm^−1^ (aromatic skeleton C–C stretching), 1456 cm^−1^ (asymmetric bending of CH_3_), and 1271 cm^−1^ (guaiacyl ring breathing with carbonyl stretching). Comparison analysis of each pretreated biomass in Entries 1–4 (Figure 8) shows that the biomass fractionation was successfully attained. The peak chart in Entry 4 did not indicate a peak related to lignin, and the retrieved material was identified as a lignin. Based on the above results, our developed pretreatment (Entry 4) can effectively break the network among the polymeric matrix and appears to be a more feasible approach for cellulose and lignin extraction.

### Biocatalytic conversion of a high-solid loading of biomass to ethanol after pretreatment with IL

Working with a high-solid loading of a target substrate is an advantage for bioethanol production because it could increase final product concentrations and plant productivity while lowering energy and water input. Therefore, many studies have been focused on new process construction that utilize high-solid loading. For this purpose, a digestible and cellulose-rich material is ideal. However, biomass that has not been pretreated has a typically low degree of digestibility and a high lignin content (30 to 50%). Therefore, the removal of lignin in pretreatment is a critical step for obtaining a high ethanol concentration.

Using the pretreatment developed in this study, lignocellulosic biomass could be fractionated to digestible cellulosic pulp (cellulose content over 84%), which shows promise for the high concentration production of chemicals and fuels. Therefore, the high degree of glucose and ethanol produced from our pretreated wood biomass was investigated. For fermentation, more than 4% (40 g/L) of ethanol production is desirable for downstream processing (distillation). Without this, the energy requirement would be too high when using a low ethanol concentration [38,39].

**Figure 9:**
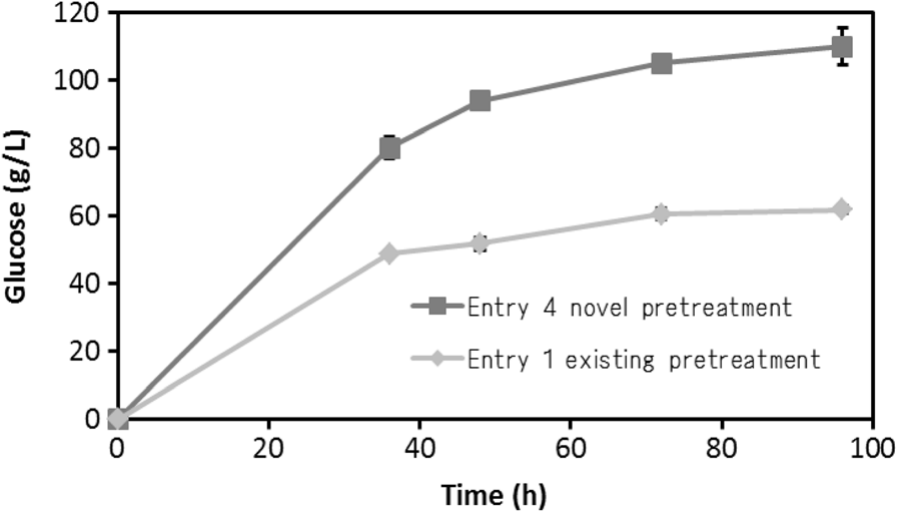
**Enzymatic hydrolysis of the novel pretreated biomass and existing pretreated biomass at high-solid loading.** Each data were averages of two independent experiments.

These background situations suggested that the attainment of a high concentration of fermentable sugar would require a high-solid loading. Therefore, the ability of of pretreated biomass to hydrolyze (Entry 4) was investigated under conditions of high loading (Figure 9). Since the enzymatic hydrolysis of biomass becomes dramatically weak under high-solid loading [40], the enzyme loading was increased to 20 filter paper units (FPU)/g-biomass. The glucose from softwood reached 110 g/L in the hydrolysate after 96 hours of saccharification. In addition, untreated biomass and a previously reported pretreatment combination (Entry 1) were tested using the same procedure for comparison. The untreated biomass did not liquefy, but the pretreated biomass exhibited degradation. However, glucose concentration was lower (62 g/L, at 96 hours) compared with that of Entry 4. Therefore, our developed method is effective for high-solid loading saccharification, and could possibly be applied to the high concentrate fermentation of ethanol through the use of a microbe.

**Figure 10:**
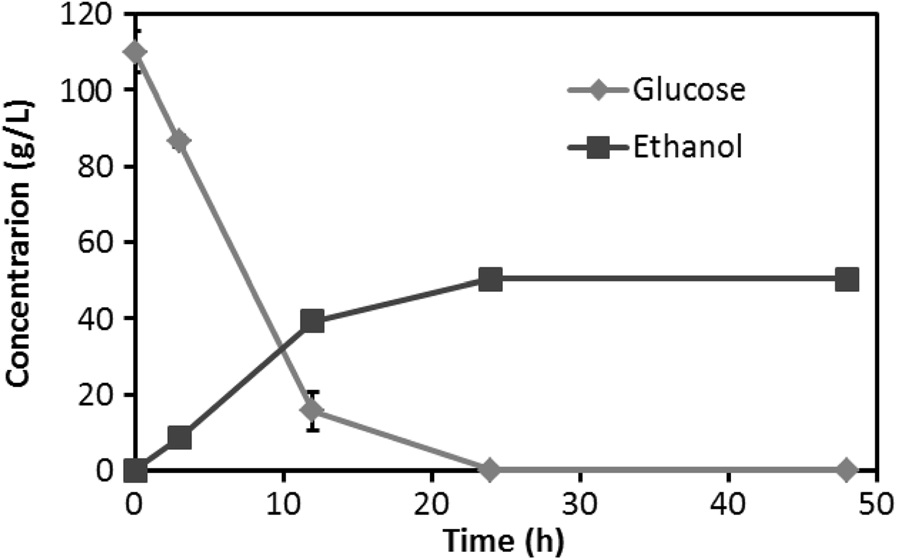
**The production of ethanol and decrease in glucose concentration during fermentation of 96 hours hydrolysates.** Each data were averages of two independent experiments.

After 96 hours of saccharification, as shown in Entry 4 (Figure 8), we added yeast to a hydrolysates solution and fermentation was conducted at 30°C (Figure 10). The glucose concentration decreased rapidly with fermentation time. After 24 hours of fermentation, the glucose was completely assimilated and the concentration of the ethanol achieved a plateau of 50 g/L. Obviously, since the result surpassed 40 g/L of ethanol production, a high level of ethanol production from softwood was achieved by the proposed method.

## Conclusions

Pretreatment is important for the conversion of low-cost lignocellulosic biomass into sugars and other chemicals. Our findings revealed that changing the pretreatment solvent and anti-solvent increased the pretreatment effectiveness. The novel pretreatment we established eased the process of biomass biodegradation and produced lignin as a by-product after the evaporation of the acetone. A high concentration of glucose (110 g/L) and ethanol production (50 g/L) was achieved with a high-solid loading by our proposed pretreatment combination.

Our goal was to overcome the high cost of the acid-catalyzed pretreatment of ILs. The cost of ILs has always been driven up by the high cost of the components and by the expensive purification process needed to produce them, which has always been a major concern [41]. Therefore, we must find other ILs that could be derived from ‘low-cost’ starting materials without purification, and integrate these into the findings in this paper to determine a novel IL that could be pretreated on an industrial scale [42].

## Material and Methods

### Materials

Japanese cedar in particle sizes of less than 200 μm were purchased from Toyota Motor Corporation (Miyoshi, Japan). The cellulose content of the biomass sample used in the present study was determined by chemical analysis conducted by Toray Techno Co., Ltd. (Ohtsu, Japan), and the results showed a glucose content of 40.8 wt% (cellulose content was 36.72 wt%, as calculated by anhydro correction factors of 162 /180). The moisture content of the untreated biomass was 7.0% based on oven-dry weight measurement. The biomass was stored in a desiccator at room temperature. The 1-butyl-3-methylimidazolium chloride ([BMIM] Cl, purity ≥98%) was purchased from Sigma-Aldrich (St. Louis, Missouri, United States), and the 1-ethyl-3-methylimidazolium acetate ([EMIM] OAc, purity >95%) was purchased from IoLiTec (Heilbronn, Germany). Commercial cellulase (Cellic CTec2) was kindly gifted from Novozymes Japan, Ltd (Chiba, Japan). All other chemicals used in this study were of analytical reagent grade. The yeast strain *S. cerevisiae* MN8140X/TF-TF was bred in our laboratory, and was used for the ethanol fermentation [43].

### Pretreatment of biomass

The Japanese cedar was pretreated with IL and catalysts. Typically, biomass (100 mg) is dissolved in a pretreatment solvent (1,000 mg, see Results and discussion for a more elaborate description) with magnetic stirring (set at 120 rpm) at 130°C for 30 minutes using an organic synthesizer, ChemStation PPS-CTRL (EYELA, Tokyo, Japan). The vessel was covered with a lid to avoid water loss. After the pretreatment, the samples were cooled to room temperature and mixed with 10 mL of an anti-solvent such as deionized water. Following that, this suspension mixture was followed by centrifugation at 3,000 rpm for 5 minutes. To remove the IL and soluble catalysts, the precipitant was washed five times with 10 mL of deionized water. Finally, we used 20 g/L of the starting biomass solution to dilute the measuring cylinder to a total of 5 mL. In order to obtain enough material for compositional analysis and enzymatic hydrolysis at high-solid loading and fermentation, the pretreatment experiments were scaled up several-fold. The resultant regenerated biomass was lyophilized for 24 hours.

### Saccharification to evaluate each pretreatment

In a 96 deepwell (Corning Inc., New York, United States), we mixed 500 μL of 20 g/L starting biomass suspension (final concentration: 10 g/L), 0.2 μL of CTec2 (2 FPU/g-starting biomass), 100 μL of 500 mM acetate buffer (pH 5.0, final concentration: 50 mM) and 399.8 μL of distilled water (DW). Since the optimal pH and temperature of Cellic CTec2 was approximately pH 5.0 to 5.5 and 45 to 50°C, respectively, the saccharification conditions were set accordingly (pH 5.0 and 50°C). The enzymatic saccharification was carried out at 1,500 rpm in a Deep Well Maximizer (TAITEC, Koshigaya, Japan), and the released glucose concentration was measured using a glucose kit (Wako, Osaka, Japan).

### Analysis of biomass composition

The cellulose contents of the biomass were determined using National Renewable Energy Laboratory (NREL) methods [44] with minor modifications. Briefly, 0.3 g of the sample (after drying) were mixed with 3 mL of a 72% (v/v) H_2_SO_4_ aqueous solution for 1 hour at 30°C using an organic synthesizer with magnetic stirring (120 rpm). The mixture was diluted with 84 mL of water and autoclaved at 121°C for 1 hour. The amount of glucose in the hydrolysate was determined via a glucose kit. The amount of cellulose was calculated from the glucose content multiplied by anhydro correction factors of 162/180. In this study, the amount of hemicellulose was regarded as the residue from the biomass excluding lignin and cellulose.

The lignin content of the substrate was measured via the thioglycolic acid lignin method [45]. We extracted 20 mg dried samples once with 2 mL water, centrifuged them for 10 minutes at 16,100 × g at room temperature (RT), and the supernatant was discarded. Next, the pellet was extracted with 1.8 mL methanol at 60°C for 20 minutes, and the process was repeated once. The pellet was dried *in vacuo* and weighed, and 1 mL of 3 N HCl and 0.1 mL of thioglycolic acid were added, followed by heating at 80°C for 3 hours. After centrifugation, the supernatant was removed and the pellet was vortexed for 30 seconds in 1 mL distilled water. After centrifugation at 16,100 × g for 10 minutes at RT, the supernatant was discarded and the pellet was re-suspended in 1 mL of 1 N sodium hydroxide (NaOH), then shaken vertically at 80 rpm for 16 hours at RT. The tubes were centrifuged at 16,100 × g for 10 minutes at RT, and the supernatant (1 mL) was transferred to fresh 1.5 mL tubes and acidified with 0.2 mL of concentrated HCl. After chilling the tubes at 4°C for 4 hours, they were centrifuged at 16,100 g for 10 minutes at RT. The supernatant was removed and the pellet was dissolved in 1 mL 1 N NaOH. After a 50-fold dilution with 1 N NaOH, the solution was submitted to spectrophotometric measurement at 280 nm. The lignin amount was calculated by using a previously reported calibration plot [45].

### Characterization of the wood and regenerated biomass using FT-IR

For the FTIR analysis of biomass, 10 mg of a solid sample was mixed with 50 mg of spectroscopic-grade KBr, ground, and pressed to produce transparent pellets using a laboratory mill and press (QM-1 and QP-1, Chromato Science, Osaka, Japan). Analyses were conducted using an FTIR spectrometer (Nexus 470, Thermo Fisher Scientific K.K., Yokohama, Japan) over a range of 4,000 to 400 cm^−1^ with a resolution of 2 cm^−1^ and 32 scans per sample. The background spectrum of pure KBr was subtracted from the spectrum of the sample.

### Saccharification and fermentation at high-solid loading

Saccharification was performed in a 50 mL polypropylene tube (Corning Inc., New York, United States), which was set in a heat block (Thermo Block Rotator SN-06BN; Nissinrika Co., Tokyo, Japan). The vessel was closed with a silicon plug (AS ONE, Osaka, Japan), into which a hole was bored using a disposable needle (Terumo Corp., Tokyo, Japan). In the vessel, 1 g of pretreated biomass (after drying) was mixed with 5.2 mL of fermentation medium containing 100 mM citrate buffer (pH 5.5) and CTec2 at a concentration of 20 FPU/g-pretreated biomass, by axially rotating the vessel at 35 rpm under a controlled temperature of 50°C.

The yeast was aerobically cultivated for 36 hours at 30°C and 150 rpm in 7 mL of mixture of yeast extract 10 g/L, polypepton 20 g/L, and glucose 20 g/L (YPD) medium. The yeast cells were collected by centrifugation at 3,000 rpm for 5 minutes and washed twice with distilled water. Fermentation was initiated by adding the 100 μL yeast solution for a final OD600 of 20. During fermentation, the temperature was maintained at 30°C to adjust the yeast activity. Glucose and ethanol were analyzed via a glucose kit and gas chromatography (GC), respectively.

### Gas chromatography analysis for ethanol

The supernatant of the culture medium was diluted with acetone to precipitate the components of the culture. After centrifugation, the supernatant was collected in a glass vial for GC analysis. The ethanol concentration was determined using a GC system (model GC-2010 Plus, Shimadzu, Kyoto, Japan) equipped with a flame ionization detector (FID) and a DB-FFAP capillary column (0.25 mm ID, Agilent Technologies, Santa Clara, California, United States). The column temperature was initially maintained at 40°C for 1 minute and then increased to 170°C at 10°C per min^−1^, then again to 230°C at 15°C per min^−1^, and finally maintained at this temperature for 5 minutes.

## Abbreviations

[BMIM] Cl: 1-butyl-3-methylimidazolium chloride
[EMIM] OAc: 1-ethyl-3-methylimidazolium acetate
DMSO: Dimethyl sulfoxide
IL: Ionic liquid
NREL: National renewable energy laboratory
SHF: Separate hydrolysis and fermentation
SSF: Simultaneous saccharification and fermentation
FTIR: Fourier transform infrared spectroscopy

## References

[1] Climent MJ, Corma A, Iborra S (2014). Conversion of biomass platform molecules into fuel additives and liquid hydrocarbon fuels. Green Chem.

[2] Hasunuma T, Kondo A (2012). Consolidated bioprocessing and simultaneous saccharification and fermentation of lignocellulose to ethanol with thermotolerant yeast strains. Process Biochem.

[3] Adsul MG, Singhvi MS, Gaikaiwari SA, Gokhale DV (2011). Development of biocatalysts for production of commodity chemicals from lignocellulosic biomass. Bioresour Technol.

[4] Agbor VB, Cicek N, Sparling R, Berlin A, Levin DB (2011). Biomass pretreatment: fundamentals toward application. Biotechnol Adv.

[5] Da Silva AS, Inoue H, Endo T, Yano S, Bon EPS (2010). Milling pretreatment of sugarcane bagasse and straw for enzymatic hydrolysis and ethanol fermentation. Bioresour Technol.

[6] Varga E, Réczey K, Zacchi G (2004). Optimization of steam pretreatment of corn stover to enhance enzymatic digestibility. Appl Biochem Biotechnol.

[7] Hsu T-C, Guo G-L, Chen W-H, Hwang W-S (2010). Effect of dilute acid pretreatment of rice straw on structural properties and enzymatic hydrolysis. Bioresour Technol.

[8] Wan C, Li Y (2010). Microbial pretreatment of corn stover with Ceriporiopsis subvermispora for enzymatic hydrolysis and ethanol production. Bioresour Technol.

[9] Alinia R, Zabihi S, Esmaeilzadeh F, Kalajahi JF (2010). Pretreatment of wheat straw by supercritical CO2 and its enzymatic hydrolysis for sugar production. Biosyst Eng.

[10] Zhu S, Wu Y, Yu Z, Chen Q, Wu G, Yu F, Wang C, Jin S (2006). Microwave-assisted alkali pre-treatment of wheat straw and its enzymatic hydrolysis. Biosyst Eng.

[11] Ma F, Yang N, Xu C, Yu H, Wu J, Zhang X (2010). Combination of biological pretreatment with mild acid pretreatment for enzymatic hydrolysis and ethanol production from water hyacinth. Bioresour Technol.

[12] Taherzadeh MJ, Karimi K (2008). Pretreatment of lignocellulosic wastes to improve ethanol and biogas production: a review. Int J Mol Sci.

[13] Haghighi Mood S, Hossein Golfeshan A, Tabatabaei M, Salehi Jouzani G, Najafi GH, Gholami M, Ardjmand M (2013). Lignocellulosic biomass to bioethanol, a comprehensive review with a focus on pretreatment. Renew Sustain Energy Rev.

[14] Swatloski RP, Spear SK, Holbrey JD, Rogers RD (2002). Dissolution of cellose with ionic liquids. J Am Chem Soc.

[15] Fort DA, Remsing RC, Swatloski RP, Moyna P, Moyna G, Rogers RD (2007). Can ionic liquids dissolve wood? processing and analysis of lignocellulosic materials with 1-n-butyl-3-methylimidazolium chloride. Green Chem.

[16] Sun N, Rahman M, Qin Y, Maxim ML, Rodríguez H, Rogers RD (2009). Complete dissolution and partial delignification of wood in the ionic liquid 1-ethyl-3-methylimidazolium acetate. Green Chem.

[17] Fu D, Mazza G (2011). Aqueous ionic liquid pretreatment of straw. Bioresour Technol.

[18] Cammarata L, Kazarian SG, Salter PA, Welton T (2001). Molecular states of water in room temperature ionic liquids. Phys Chem Chem Phys.

[19] Zhang Z, O’Hara IM, Doherty WOS (2012). Pretreatment of sugarcane bagasse by acid-catalysed process in aqueous ionic liquid solutions. Bioresour Technol.

[20] Zhang Z, O’Hara IM, Doherty WOS (2013). Effects of pH on pretreatment of sugarcane bagasse using aqueous imidazolium ionic liquids. Green Chem.

[21] Diedericks D, van Rensburg E, García-Aparicio MDP, Görgens JF (2012). Enhancing the enzymatic digestibility of sugarcane bagasse through the application of an ionic liquid in combination with an acid catalyst. Biotechnol Prog.

[22] Nguyen Q, Tucker M (2002). Dilute acid/metal salt hydrolysis of lignocellulosics. US Pat.

[23] Yan Y, Li T, Ren Z, Li G (1996). A study on catalytic hydrolysis of peat. Bioresour Technol.

[24] Stärk K, Taccardi N, Bösmann A, Wasserscheid P (2010). Oxidative depolymerization of lignin in ionic liquids. ChemSusChem.

[25] Liu L, Sun J, Cai C, Wang S, Pei H, Zhang J (2009). Corn stover pretreatment by inorganic salts and its effects on hemicellulose and cellulose degradation. Bioresour Technol.

[26] Sannigrahi P, Kim DH, Jung S, Ragauskas A (2011). Pseudo-lignin and pretreatment chemistry. Energy Environ Sci.

[27] Da Costa Lopes AM, João KG, Morais ARC, Bogel-Łukasik E, Bogel-Łukasik R (2013). Ionic liquids as a tool for lignocellulosic biomass fractionation. Sustain Chem Process.

[28] Viell J, Marquardt W (2011). Disintegration and dissolution kinetics of wood chips in ionic liquids. Holzforschung.

[29] Wang X, Li H, Cao Y, Tang Q (2011). Cellulose extraction from wood chip in an ionic liquid 1-allyl-3-methylimidazolium chloride (AmimCl). Bioresour Technol.

[30] Berlin A, Balakshin M, Gilkes N, Kadla J, Maximenko V, Kubo S, Saddler J (2006). Inhibition of cellulase, xylanase and beta-glucosidase activities by softwood lignin preparations. J Biotechnol.

[31] Bokinsky G, Peralta-Yahya PP, George A, Holmes BM, Steen EJ, Dietrich J, Lee TS, Tullman-Ercek D, Voigt CA, Simmons BA, Keasling JD (2011). Synthesis of three advanced biofuels from ionic liquid-pretreated switchgrass using engineered Escherichia coli. Proc Natl Acad Sci U S A.

[32] Cox BJ, Ekerdt JG (2013). Pretreatment of yellow pine in an acidic ionic liquid: extraction of hemicellulose and lignin to facilitate enzymatic digestion. Bioresour Technol.

[33] Kadla J, Kubo S, Venditti R, Gilbert R, Compere A, Griffith W (2002). Lignin-based carbon fibers for composite fiber applications. Carbon N Y.

[34] da Silva EAB, Zabkova M, Araújo JD, Cateto CA, Barreiro MF, Belgacem MN, Rodrigues AE (2009). An integrated process to produce vanillin and lignin-based polyurethanes from Kraft lignin. Chem Eng Res Des.

[35] Labbé N, Rials TG, Kelley SS, Cheng Z-M, Kim J-Y, Li Y (2005). FT-IR imaging and pyrolysis-molecular beam mass spectrometry: new tools to investigate wood tissues. Wood Sci Technol.

[36] Bilba K, Ouensanga A (1996). Fourier transform infrared spectroscopic study of thermal degradation of sugar cane bagasse. J Anal Appl Pyrolysis.

[37] Casas A, Alonso MV, Oliet M, Rojo E, Rodríguez F (2012). FTIR analysis of lignin regenerated from Pinus radiata and Eucalyptus globulus woods dissolved in imidazolium-based ionic liquids. J Chem Technol Biotechnol.

[38] Zacchi G (2005). Hydrolysis of Biomass for fuel ethanol production.

[39] Zhao X, Song Y, Liu D (2011). Enzymatic hydrolysis and simultaneous saccharification and fermentation of alkali/peracetic acid-pretreated sugarcane bagasse for ethanol and 2,3-butanediol production. Enzyme Microb Technol.

[40] Kristensen JB, Felby C, Jørgensen H (2009). Yield-determining factors in high-solids enzymatic hydrolysis of lignocellulose. Biotechnol Biofuels.

[41] Tadesse H, Luque R (2011). Advances on biomass pretreatment using ionic liquids: an overview. Energy Environ Sci.

[42] Olivier-Bourbigou H, Magna L, Morvan D (2010). Ionic liquids and catalysis: recent progress from knowledge to applications. Appl Catal A Gen.

[43] Sanda T, Hasunuma T, Matsuda F, Kondo A (2011). Repeated-batch fermentation of lignocellulosic hydrolysate to ethanol using a hybrid Saccharomyces cerevisiae strain metabolically engineered for tolerance to acetic and formic acids. Bioresour Technol.

[44] Sluiter A, Hames B, Ruiz R, Scarlata C, Sluiter J, Templeton D, Nrel DC (ᅟ). Determination of structural carbohydrates and lignin in biomass determination of structural carbohydrates and lignin in biomass. Technical report: NREL/TP-510-42618.

[45] Suzuki S, Suzuki Y, Yamamoto N, Hattori T, Sakamoto M, Umezawa T (2009). High-throughput determination of thioglycolic acid lignin from rice. Plant Biotechnol.

